# Tobacco Use Among Racial and Ethnic Population Subgroups of Adolescents in the United States

**Published:** 2006-03-15

**Authors:** Ralph S Caraballo, Sue Lin Yee, Terry F Pechacek, Rosemarie Henson, Joseph C Gfroerer

**Affiliations:** Office on Smoking and Health, National Center for Chronic Disease Prevention and Health Promotion, Centers for Disease Control and Prevention; National Center for Chronic Disease Prevention and Health Promotion, Centers for Disease Control and Prevention, Atlanta, Ga; National Center for Chronic Disease Prevention and Health Promotion, Centers for Disease Control and Prevention, Atlanta, Ga; National Center for Chronic Disease Prevention and Health Promotion, Centers for Disease Control and Prevention, Atlanta, Ga; Office of Applied Studies, Substance Abuse and Mental Health Services Administration, Rockville, Md

## Abstract

**Introduction:**

Limited data on cigarette smoking among population subgroups hinder the development and implementation of intervention strategies for those subgroups. Because of small sample sizes or inadequate study formats, cigarette smoking among youths has been studied mostly among broad racial or ethnic categories (e.g., Asian, Hispanic) instead of subgroups (e.g., Vietnamese, Cuban). The objective of this study was to evaluate cigarette smoking among U.S. youths by racial and ethnic subgroups.

**Methods:**

The study used a nationally representative sample of youths aged 12 to 17 years who participated in the National Survey on Drug Use and Health in 1999, 2000, or 2001. Outcomes measured include prevalence of cigarette smoking, mean age of smoking initiation, and susceptibility to start smoking.

**Results:**

The prevalence of smoking among youths aged 12 to 17 years varied among racial and ethnic subgroups, ranging from 27.9% for American Indians and Alaska Natives to 5.2% for Japanese. Among youths aged 12 to 17 years, the age of smoking initiation ranged from 11.5 years  (American Indians and Alaska Natives) to 13.2 years (Japanese); the overall mean age of initiation was 12.3 years. White and African American youths were the only groups that showed a significant sex difference in age of initiation among all 14 subgroups; white and African American boys initiated smoking a few months earlier than white and African American girls. One of every four never-smokers aged 12 to 17 years was classified as susceptible to becoming a smoker.

**Conclusion:**

The prevalence of cigarette smoking among youths varies widely by racial and ethnic subgroup. There is a need for sustained, culturally appropriate interventions to prevent and control cigarette smoking among youths, particularly within racial and ethnic subgroups with a high prevalence of cigarette smoking.

## Introduction

One of the challenges in developing and securing resources for the implementation of effective programmatic and policy interventions for population subgroups that have disproportionately high rates of tobacco use or, specifically, cigarette smoking is the limited amount of data on those subgroups. Because of small sample sizes or inadequate study formats, tobacco use among youths was only studied among broad racial or ethnic categories in the 1998 Surgeon General's report (1) and in several school-based surveys (e.g., the Youth Risk Behavior Survey, the National Youth Tobacco Survey, the Monitoring the Future Survey) (2-4). The 1998 Surgeon General's report examined tobacco use among the broad racial and ethnic categories of African Americans, American Indians and Alaska Natives, Asian Americans and Pacific Islanders, and Hispanics. There is wide recognition among tobacco control researchers and public health practitioners, however, that aggregate data on these four racial and ethnic categories may not fully describe important differences in smoking prevalence, tobacco industry marketing to U.S. population subgroups, and the attitudes, behaviors, and health-related knowledge of subgroups within these four broad categories (5-8).

To monitor future progress in tobacco control among youths aged 12 to 17 years in the United States, current tobacco use and cigarette smoking prevalence estimates are needed for the racial and ethnic subgroups within this age group. In this article, we provide data on smoking for girls and boys in the following 14 groups and subgroups: white, African American, American Indian or Alaska Native, Hawaiian or Other Pacific Islander, Chinese, Filipino, Japanese, Asian Indian, Korean, Vietnamese, Mexican, Puerto Rican, Central or South American, and Cuban. These racial and ethnic classifications adhere to the new standards for the collection of data on races and ethnicities within the federal statistical system (9).

## Methods

To provide data for racial and ethnic subgroups, we analyzed data from the National Survey on Drug Use and Health (NSDUH), formerly called the National Household Survey on Drug Abuse, by combining data for the years 1999, 2000, and 2001 (10,11). NSDUH is a nationwide household survey that collects information on drug use and drug abuse from a representative sample of the U.S. civilian, noninstitutionalized population aged 12 years and older. Cigarette smoking is one behavior about which NSDUH collects information. NSDUH has been conducted since 1971. In 1999, NSDUH implemented a major redesign; changes were made in both sample design and the data collection method. The national design was changed to allow estimation of state data. The data collection method was changed from a paper-and-pencil interview to a computer-assisted interview (CAI), primarily to improve the quality of NSDUH estimates.

The three surveys that we combined each used the same sample design and data collection method. The sample design consists of deeply stratified, multistage, area probability designs (10). The survey is administered through confidential, anonymous, face-to-face interviews in the household by trained interviewers using a CAI questionnaire. The tobacco use section was administered using audio computer-assisted self-interviewing (ACASI), in which the respondent reads questions on the computer screen or listens to questions through headphones and then records answers into a computer. The nationally representative sample of the surveyed population permits calculation of national estimates.

### Study population 

We included NSDUH participants aged 12 to 17 years (N = 74,207) (Table 1). The average weighted overall response rate for this age group in the combined surveys was 74.0%. This rate is the product of the weighted household screening response rate (91.4%) and the weighted individual-level response rate for selected youths (80.9%) during 1999 through 2001.

#### Demographic classification

Race and ethnicity designation was based on the respondent's self-classification. For ethnicity, respondents were asked, "Are you of Hispanic, Latino, or Spanish origin or descent?" Hispanic respondents were also asked to select a subgroup (e.g., Mexican, Puerto Rican, Central or South American, or Cuban) that best described them. For race, respondents were asked, "Which of these groups best describes you?" Response alternatives were 1) white, 2) black or African American, 3) American Indian or Alaska Native, 4) Native Hawaiian, 5) Other Pacific Islander, 6) Asian, and 7) Other. Asians were also asked to select a subgroup (e.g., Chinese, Filipino, Japanese, Asian Indian, Korean, or Vietnamese) that best described them. Because of small sample size, the subgroups Native Hawaiian and Other Pacific Islander were combined. For this article, all Hispanics are included in the Hispanic group, regardless of race. All other categories of race and ethnicity exclude Hispanics. For simplicity, we refer to non-Hispanic whites as whites, and non-Hispanic blacks as African Americans.

#### Tobacco-related variables

The tobacco section of the NSDUH questionnaire has 43 items on the use of cigarettes, chewing tobacco, snuff (dip), cigars, or pipes. We limited our analysis to cigarette smoking, because for the most part, there was low use of other tobacco products among the survey population. A current cigarette smoker is defined as anyone who answered yes to the question, "During the past 30 days, have you smoked part or all of a cigarette?" A previous smoker is defined as anyone who reported having smoked one or more cigarettes in his or her lifetime but not in the previous month. The mean age of smoking initiation was calculated by using data from youths aged 12 to 17 years who reported ever using cigarettes. The question asked was, "How old were you the first time you smoked part or all of a cigarette?" Susceptibility to start smoking among self-reported nonsmokers was determined by the following two questions: 1) "If one of your best friends offered you a cigarette, would you smoke it?" and 2) "At any time during the next 12 months, do you think that you will smoke a cigarette?" Possible answers were definitely yes, probably yes, probably not, definitely not. Those who answered "definitely not" to both questions were classified as nonsusceptible; those who answered any other combination of choices were considered to be susceptible to start smoking.

### Statistical analysis 

Cross-tabulation of the variables of interest (cigarette smoking, susceptibility to start smoking) by race and ethnicity was performed. The average age of smoking initiation was also calculated. Confidence intervals (95%) were calculated to provide information on sampling errors. Differences in estimates were considered statistically significant when confidence intervals did not overlap. No multiple comparisons testing (e.g., Tukey, Sheffé Bonferroni) was performed to determine if any pair of estimates was statistically different. All prevalence measures and confidence intervals were estimated using SUDAAN version 6.40 (Research Triangle Institute, Research Triangle Park, NC) to account for the complex survey design. Survey weights were used to account for different probabilities of selection within strata.

## Results

There were more previous than current smokers in all racial and ethnic groups, except for American Indians and Alaska Natives, for whom the percentage of previous smokers was about the same as the percentage of current smokers (Table 2). The percentage of previous smokers ranged from 29.4% (American Indians and Alaska Natives) to 10.5% (Asian Indians). Among Asian subgroups, Filipinos (20.3%) and Vietnamese (18.5%) had about twice as many previous smokers as Asian Indians (10.5%). The only subgroup that had more current and previous smokers than never-smokers was American Indians and Alaska Natives.

The prevalence of current cigarette smoking varied widely, ranging from as high as 27.9% among American Indians and Alaska Natives to as low as 5.2% among Japanese (Table 3). Using the overall prevalence of current smoking (13.8%) as the referent group in statistical comparisons, we found that American Indians and Alaska Natives (27.9%) and whites (16.0%) had a higher prevalence of current smoking, whereas most other racial and ethnic subgroups had a lower prevalence. Results for Hawaiian and Other Pacific Islanders, Asian Indians, Koreans, and Cubans were not statistically different from the national estimate (Table 3). Among Asian subgroups, the prevalence of current cigarette smoking ranged from 10.6% among Koreans to 5.2% among Japanese; however, none of these differences was significant. Among Hispanic subgroups, the prevalence of current cigarette smoking did not differ significantly, ranging from 12.4% among Cubans to 9.6% among Central or South Americans. As a combined group, Asians (8.1%) had a lower prevalence of current cigarette smoking in this age group than Hispanics (10.8%).

Comparisons by sex for each of the racial and ethnic subgroups showed no differences in current cigarette smoking, except for whites and African Americans. White girls (17.2%) had a slightly higher prevalence of current smoking than white boys (14.9%), and African American boys (8.2%) had a slightly higher prevalence of current smoking than African American girls (5.9%) (Table 3). Compared with national estimates for boys (13.3%), American Indian and Alaska Native (29.5%) and white (14.9%) boys had a higher prevalence of current smoking, whereas African American (8.2%), Chinese (6.3%), and Filipino (5.8%) boys had a lower prevalence. Results for all other subgroups were not significantly different from the national estimate for boys (Table 3). Compared with national estimates for girls (14.2%), American Indian and Alaska Native (26.3%) and white (17.2%) girls had a higher prevalence of current smoking, whereas African American (5.9%), Chinese (5.4%), Mexican (10.6%), Puerto Rican (10.4%), and Central or South American (9.3%) girls had a lower prevalence (Table 3).

Among youths aged 12 to 17 years, the mean age of initiation ranged from 11.5 years (American Indians and Alaska Natives) to 13.2 years (Japanese) (Table 4). Whites and African Americans were the only groups that showed a statistically significant sex difference in age of initiation; white and African American boys started smoking a few months earlier than white and African American girls (Table 4). Among boys, the mean age of smoking initiation was 12.1 years. The mean age of initiation for American Indian and Alaska Native boys (11.4 years) was younger than the national mean for boys. Among girls, the mean age of initiation was 12.5 years of age. Only American Indian and Alaska Native girls had a younger age of initiation (11.7 years). Chinese (13.5 years), Japanese (13.8 years), and Asian Indian (13.8 years) girls were older at smoking initiation than the national mean.

A wide range in susceptibility to start smoking was observed among youths aged 12 to 17 years who had never smoked (Table 5). Overall, one of four (24.6%) never-smokers was susceptible to start smoking. African American (27.1%) and Mexican (31.5%) never-smokers seemed to be more susceptible to start smoking than the national average; whites (23.0%), Japanese (12.2%), and Asian Indian (16.6%) never-smokers seemed to be less susceptible to start smoking. Among boys, African American (27.9%) and Mexican (33.2%) never-smokers seemed to be more susceptible to start smoking than the national average (25.2%), and white (23.3%) and Asian Indian (14.7%) never-smokers seemed to be less susceptible to start smoking. Finally, among girls, African American (26.4%) and Mexican (29.9%) never-smokers seemed to be more susceptible to start smoking than the national average (24.0%).

## Discussion

This study shows disparities in the prevalence of cigarette smoking, mean age at smoking initiation, and susceptibility to start smoking among some of the 14 population subgroups studied. The study also shows some sex differences. Factors associated with susceptibility to start smoking (preparatory stage), smoking initiation (trying stage), daily smoking (regular use), and prevalence of cigarette smoking (occasional and regular use) are complex and are not necessarily exactly the same for any population subgroup. Some of these factors may vary among racial or ethnic subgroups (12,13); others are common. Factors that may affect several stages of smoking (i.e., preparatory, trying, experimental, and regular use) are smoking by peers, siblings, parents, or caregivers; social pressure to smoke by such people; young age of the potential smoker; problem behaviors in the youth; the youth's receptivity to tobacco advertisement and promotion; cigarette price; acculturation; attitudes toward smoking; perceived benefits of smoking; and other factors (12-24).

Findings in this study that merit further discussion are the following: 1) disparities in susceptibility to start smoking among never-smokers, 2) disparities in age of smoking initiation, 3) limitations of this study, and 4) implications of these findings.

This article clearly shows that of all groups or subgroups studied, American Indian or Alaska Native is the group at highest risk of cigarette smoking. They have the highest prevalence of smoking, highest previous use of cigarettes, and are more likely to have ever smoked. More than 25% of American Indian or Alaska Native never-smokers are susceptible to start smoking. This group also starts smoking at the youngest age, and both boys and girls in this group have a high prevalence of cigarette smoking. Previous reports have found that American Indian or Alaska Native youths and adults have the highest prevalence of cigarette smoking (1,12,25-28). Thus, this group deserves special attention with regard to prevention and cessation interventions.

Another group that deserves special attention is white youths. They also have a high prevalence of smoking compared with most other racial or ethnic groups or subgroups, although not as high as the American Indian or Alaska Natives group. Almost 40% of white youths have ever smoked a cigarette. In this age group, girls have a slightly higher prevalence of smoking than boys.

African Americans have a low prevalence of cigarette smoking compared with many of the other racial or ethnic groups or subgroups. Less than 10% of African Americans aged 12 to 17 years were current cigarette smokers during 1999 through 2001, and about 75% of them had never smoked. African Americans are only one of two groups to show a significant sex difference in smoking: boys have a higher prevalence of smoking than girls, and boys also initiate smoking a few months earlier than girls. Unfortunately, the low smoking prevalence among African American youths is not seen among African American adults. After American Indian or Alaska Native, African American and white adults have the highest overall prevalence of cigarette smoking.

Hawaiian or Other Pacific Islander youths have a higher prevalence of cigarette smoking than Asians. Also, their age of initiation (11.8 years) is younger than the age of initiation for Asian subgroups (ranging from 12.1 to 13.2 years), although the age difference is not statistically significant. It is known that there are distinct variations in cigarette smoking patterns and behaviors between Hawaiians or Other Pacific Islanders and Asians (1), with Hawaiians or Other Pacific Islanders having a higher prevalence of cigarette smoking than Asians overall.

Many of the Asian subgroups have a lower prevalence of smoking than other non-Asian groups or subgroups. For example, Chinese, Filipino, Japanese, and Vietnamese subgroups have a lower prevalence of cigarette smoking than the national average. Also, compared to the national estimate, Asian Indians and Koreans have a lower prevalence of previous cigarette smoking.

Among Hispanic subgroups, the prevalence of cigarette smoking is relatively low, about 10%. No sex differences in cigarette smoking were found among any Hispanic subgroups. However, Puerto Rican boys and girls who never smoked seemed to be less susceptible to start smoking than Mexican boys and girls. Even though the prevalence of cigarette smoking among Mexicans, Puerto Ricans, Central or South Americans, and Cubans is not as high as that of some other groups, differences in smoking have been found among Hispanic adult subgroups. NSDUH shows that Puerto Ricans in general have a higher prevalence of smoking (32.5%) than Mexicans (24.6%), Central or South Americans (23.1%), and Cubans (21.1%) (S.L.Y., unpublished data, 2005).

Smoking initiation for youths aged 12 to 17 years occurs primarily in early adolescence. The younger youths are when they begin smoking, the more likely they are to smoke as adults, and the less likely they are to quit. This potentially lengthens the period of time of smoking, thus increasing the likelihood of developing smoking-related chronic diseases (12). Nicotine addiction further heightens the likelihood that adolescent cigarette users will continue to use cigarettes regularly when they are adults.

The findings in this study are subject to at least four limitations. First, NSDUH surveys are conducted in English; no surveys are conducted in native languages (e.g., Mandarin, Japanese). Thus, smoking prevalence for some population subgroups may be underestimated. Second, the precision of smoking prevalence estimates for certain population subgroups is low, especially when reported by sex. Differences in prevalence between boys and girls and among racial and ethnic population subgroups might be missed, and estimates should be interpreted with caution. Third, no adjustments for multiple comparisons were performed to determine whether differences between any pair of estimates were statistically significant. Such differences might be significant even if confidence intervals overlap. Finally, youths who did not want their parents to know they smoked might have denied smoking during their interview. This concern is especially relevant when surveys are conducted in the households of participants (12). However, the level of privacy (parental proximity) during the interview was assessed by the interviewers. The percentage of youth interviews with "complete privacy" or only "minor distractions" was generally 90.0% or more by subgroup and sex. Also, data are scarce on the magnitude of underreported smoking among adolescents using the biomarker cotinine to confirm the validity of self-reports. The data available show little evidence of smoking underreporting among whites, African Americans, and Mexican Americans (29).

Although the prevalence of cigarette smoking is lower for almost all adolescent racial and ethnic subgroups compared with American Indian and Alaska Native or white youths, some of these subgroups in adulthood have a prevalence of cigarette smoking similar to the prevalence of whites (1,25,30) and may be at an even higher risk of developing and dying from smoking-related diseases (1). Several factors may contribute to this situation: among adults, lower cessation rates among some racial and ethnic subgroups than others may help to explain similarities and differences in prevalence of cigarette smoking among these subgroups (1). Also, at the population level, almost all racial and ethnic subgroups have less access than whites to culturally and linguistically appropriate antitobacco information, educational materials, media messages, and cessation services (1). Moreover, racial and ethnic population subgroups have historically been and continue to be the target of intensive tobacco industry marketing efforts, including sponsorships of cultural events, funding of culture-specific organizations and issues, and other outreach efforts (1).

In conclusion, cigarette smoking is taking a major toll on the health of racial and ethnic population subgroups. Many of the chronic diseases that plague the American public, such as cardiovascular disease, lung disease, and many cancers, are related to cigarette smoking and other tobacco use. Thus, preventing tobacco use among youths from racial or ethnic subgroups is critical to ending the epidemic of tobacco use in the United States and reducing disparities in the burden of tobacco-related disease. Sustaining strong local and state comprehensive tobacco control programs is essential if we are to be successful in decreasing tobacco use among racial and ethnic population subgroups with a high smoking prevalence and preventing increases in tobacco use among racial and ethnic population subgroups with low tobacco use. We need to focus our prevention resources on launching effective and culturally competent initiatives that include 1) increasing capacity for education, program development, and research opportunities; and 2) strengthening the policy infrastructure to increase smoke-free environments within racial and ethnic communities. By investing in these initiatives to address the diverse needs of population subgroups, we have a tremendous opportunity to avert the development of tobacco-related diseases. The public health movement against tobacco use can declare victory when people from all racial and ethnic subgroups no longer use tobacco products.

## Figures and Tables

**Table 1:** Sample Sizes of U.S. Youths Aged 12 to 17, by Race and Ethnicity, National Survey on Drug Use and Health, 1999–2001

**Race and Ethnicity**	**Total**	**Boys**	**Girls**
**Overall^a^**	74,207	37,492	36,715

**Non-Hispanic^a^**	63,932	32,232	31,700

White	49,408	25,016	24,392
Black or African American	9,723	4,830	4,893
American Indian or Alaska Native	845	429	416
Hawaiian or Other Pacific Islander	266	141	125
Asian^a^	2,279	1,148	1,131
Chinese	334	166	168
Filipino	427	218	209
Japanese	191	91	100
Asian Indian	398	207	191
Korean	275	145	130
Vietnamese	271	129	142

**Hispanic^a^**	10,275	5,260	5,015

Mexican	6,615	3,376	3,239
Puerto Rican	1,375	714	661
Central or South American	1,293	651	642
Cuban	382	212	170

a Totals include data from respondents reporting racial and ethnic subgroups not shown as well as respondents reporting more than one subgroup. Source: Substance Abuse and Mental Health Services Administration (11).

**Table 2:** Percentage of Current, Previous, and Never-Smokers^a^ of Cigarettes Among U.S. Youths Aged 12 to 17, by Race and Ethnicity, National Survey on Drug Use and Health, 1999–2001

**Race and Ethnicity**	**Current**	**Previous**	**Never**
**Overall^b^**	13.8 (13.4-14.1)	21.3 (20.9-21.7)	64.9 (64.4-65.4)

**Non-Hispanic^b^**	14.2 (13.9-14.6)	21.3 (20.9-21.8)	64.4 (63.9-65.0)

White	16.0 (15.6-16.5)	22.2 (21.7-22.7)	61.8 (61/1-62.4)
Black or African American	7.0 (6.4-7.7)	18.3 (17.4-19.3)	74.6 (73.6-75.7)
American Indian or Alaska Native	27.9 (23.7-32.5)	29.4 (24.1-35.3)	42.8 (37.5-48.2)
Hawaiian or Other Pacific Islander	11.0 (6.4-18.2)	16.6 (11.4-23.6)	72.4 (65.2-78.6)
Asian^b^	8.1 (6.6-9.9)	15.6 (13.5-17.9)	76.3 (73.6-78.8)
Chinese	5.8 (3.3-9.9)	15.6 (11.2-21.2)	78.6 (72.3-83.8)
Filipino	7.4 (4.8-11.2)	20.3 (15.4-26.2)	72.3 (66.3-77.7)
Japanese	5.2 (2.3-11.2)	13.6 (8.2-21.7)	81.2 (72.6-87.6)
Asian Indian	8.7 (5.0-14.7)	10.5 (6.6-16.2)	80.8 (73.3-86.6)
Korean	10.6 (6.8-16.4)	12.9 (8.9-18.3)	76.5 (69.9-82.0)
Vietnamese	6.8 (3.3-13.5)	18.5 (11.8-27.8)	74.8 (64.7-82.7)

**Hispanic^b^**	10.8 (10.0-11.7)	21.3 (20.2-22.4)	67.9 (66.6-69.2)

Mexican	11.0 (10.0-12.1)	22.0 (20.7-23.3)	67.0 (65.3-68.6)
Puerto Rican	10.8 (8.7-13.3)	19.9 (16.9-23.2)	69.3 (65.8-72.6)
Central or South American	9.6 (7.4-12.3)	19.9 (17.3-22.8)	70.5 (67.0-73.8)
Cuban	12.4 (8.0-18.7)	20.4 (16.1-25.6)	67.2 (60.7-73.1)

a Current indicates cigarette smoking in past month; previous, one or more cigarettes during lifetime but not in the previous month; never, never smoked cigarettes in lifetime.

b Totals include data from respondents reporting racial and ethnic subgroups not shown as well as respondents reporting more than one subgroup. Source: Substance Abuse and Mental Health Services Administration (11).

**Table 3:** Percentage of U.S. Youths Aged 12 to 17 Who Smoked Cigarettes in Past Month, by Race, Ethnicity, and Sex, National Survey on Drug Use and Health, 1999–2001

**Race and Ethnicity**	**Total,** **% (95% Confidence Interval)**	**Boys,** **% (95% Confidence Interval)**	**Girls,** **% (95% Confidence Interval)**
**Overall^a^**	13.8 (13.4-14.1)	13.3 (12.8-13.7)	14.2 (13.8-14.7)

**Non-Hispanic^a^**	14.2 (13.9-14.6)	13.5 (13.0-13.9)	14.9 (14.5-15.4)

White	16.0 (15.6-16.5)	14.9 (14.3-15.5)	17.2 (16.6-17.8)
Black or African American	7.0 (6.4-7.7)	8.2 (7.2-9.2)	5.9 (5.1-6.8)
American Indian or Alaska Native	27.9 (23.7-32.5)	29.5 (22.8-37.3)	26.3 (20.8-32.6)
Hawaiian or Other Pacific Islander	11.0 (6.4-18.2)	7.0 (3.4-13.9)	NA^b^
Asian^a^	8.1 (6.6-9.9)	8.8 (6.7-11.6)	7.3 (5.6-9.5)
Chinese	5.8 (3.3-9.9)	6.3 (3.0-12.6)	5.4 (2.3-12.2)
Filipino	7.4 (4.8-11.2)	5.8 (3.0-11.1)	8.9 (4.9-15.7)
Japanese	5.2 (2.3-11.2)	NA^b^	NA^b^
Asian Indian	8.7 (5.0-14.7)	10.1 (4.9-19.8)	6.8 (2.9-15.1)
Korean	10.6 (6.8-16.4)	13.8 (7.9-23.0)	7.3 (3.5-14.5)
Vietnamese	6.8 (3.3-13.5)	NA^b^	8.0 (3.7-16.2)

**Hispanic^a^**	10.8 (10.0-11.7)	11.4 (10.3-12.7)	10.2 (9.1-11.4)

Mexican	11.0 (10.0-12.1)	11.4 (10.0-13.1)	10.6 (9.3-12.1)
Puerto Rican	10.8 (8.7-13.3)	11.2 (8.2-15.0)	10.4 (7.7-13.8)
Central or South American	9.6 (7.4-12.3)	9.9 (6.7-14.3)	9.3 (6.6-12.9)
Cuban	12.4 (8.0-18.7)	14.3 (7.9-24.5)	10.0 (6.0-16.0)

a Totals include data from respondents reporting racial and ethnic subgroups not shown as well as respondents reporting more than one subgroup. Source: Substance Abuse and Mental Health Services Administration (11).

b NA indicates not applicable; values are too small to report.

**Table 4:** Mean Age of Smoking Initiation Among U.S. Youths Aged 12 to 17 Who Have Ever Smoked Cigarettes, by Race and Ethnicity, National Survey on Drug Use and Health, 1999–2001

**Race and Ethnicity**	**Total, y (95% Confidence Interval)**	**Boys, y (95% Confidence Interval)**	**Girls, y (95% Confidence Interval)**
**Overall^a^**	12.3 (12.3-12.3)	12.1 (12.1-12.2)	12.5 (12.4-12.5)

**Non-Hispanic^a^**	12.3 (12.2-12.3)	12.1 (12.1-12.2)	12.4 (12.4-12.5)

White	12.3 (12.2-12.3)	12.1 (12.0-12.1)	12.4 (12.4-12.5)
Black or African American	12.4 (12.3-12.5)	12.2 (12.0-12.4)	12.7 (12.5-12.8)
American Indian or Alaska Native	11.5 (11.2-11.9)	11.4 (10.9-11.9)	11.7 (11.2-12.2)
Hawaiian or Other Pacific Islander	11.8 (10.8-12.7)	11.8 (10.0-13.5)	11.8 (11.1-12.5)
Asian^a^	12.8 (12.4-13.2)	12.8 (12.4-13.2)	12.8 (12.1-13.5)
Chinese	12.7 (11.9-13.4)	11.6 (10.3-13.0)	13.5 (12.6-14.5)
Filipino	12.7 (12.4-13.1)	12.6 (12.0-13.1)	12.9 (12.3-13.5)
Japanese	13.2 (12.2-14.2)	12.5 (10.9-14.2)	13.8 (12.8-14.8)
Asian Indian	13.1 (12.5-13.8)	12.9 (12.0-13.7)	13.8 (13.1-14.4)
Korean	13.0 (12.3-13.6)	13.5 (12.5-14.4)	12.3 (11.6-13.0)
Vietnamese	12.1 (10.2-14.0)	13.3 (12.1-14.5)	10.8 (7.7-13.9)

**Hispanic^a^**	12.5 (12.4-12.6)	12.3 (12.2-12.5)	12.6 (12.4-12.8)

Mexican	12.4 (12.3-12.5)	12.3 (12.1-12.4)	12.6 (12.4-12.8)
Puerto Rican	12.4 (12.1-12.7)	12.6 (12.2-13.0)	12.2 (11.8-12.6)
Central or South American	12.7 (12.4-13.0)	12.8 (12.3-13.2)	12.7 (12.3-13.1)
Cuban	12.5 (11.9-13.0)	12.6 (12.1-13.1)	12.3 (11.3-13.3)

a Totals include data from respondents reporting racial and ethnic subgroups not shown as well as respondents reporting more than one subgroup. Source: Substance Abuse and Mental Health Services Administration (11).

**Table 5:** Susceptibility to Start Smoking Cigarettes Among U.S. Youths Aged 12 to 17 Who Have Never Smoked, by Race and Ethnicity, National Survey on Drug Use and Health, 1999–2001

**Race and Ethnicity**	**Total,** **% (95% Confidence Interval)**	**Boys,** **%** (**95% Confidence Interval)**	**Girls,** **% (95% Confidence Interval)**
**Overall^a^**	24.6 (24.1-25.1)	25.2 (24.5-25.9)	24.0 (23.3-24.7)

**Non-Hispanic^a^**	23.5 (22.9-24.1)	23.9 (23.1-24.7)	23.1 (22.3-23.9)

White	23.0 (22.4-23.6)	23.3 (22.5-24.1)	22.8 (22.0-23.6)
Black or African American	27.1 (25.8-28.5)	27.9 (25.9-29.9)	26.4 (24.6-28.3)
American Indian or Alaska Native	27.9 (21.6-35.2)	25.4 (17.8-34.9)	30.0 (21.5-40.1)
Hawaiian or Other Pacific Islander	NA^b^	NA^b^	NA^b^
Asian^a^	20.4 (18.1-23.0)	22.7(19.1-26.7)	18.2(14.8-22.2)
Chinese	23.3 (17.7-30.0)	NA^b^	NA^b^
Filipino	21.0 (16.2-26.9)	22.1 (15.0-31.4)	19.9 (12.7-29.6)
Japanese	12.2 (7.1-20.0)	NA^b^	NA^b^
Asian Indian	16.6 (12.0-22.5)	14.7 (8.9-23.5)	18.7 (12.5-27.0)
Korean	19.2 (13.4-26.8)	23.3 (15.6-33.4)	NA^b^
Vietnamese	22.8 (15.9-31.6)	NA^b^	20.3 (12.6-30.9)

**Hispanic^a^**	29.7 (28.4-31.0)	31.3 (29.4-33.1)	28.2 (26.4-30.0)

Mexican	31.5 (30.1-33.0)	33.2 (31.0-35.5)	29.9 (27.7-32.1)
Puerto Rican	22.6 (19.4-26.2)	23.6 (19.2-28.7)	21.4 (16.9-26.7)
Central or South American	27.9 (24.3-31.8)	30.0 (25.1-35.5)	25.6 (20.7-31.2)
Cuban	27.1 (20.0-35.5)	27.3 (18.1-39.1)	NA^b^

a Totals include data from respondents reporting racial and ethnic subgroups not shown as well as respondents reporting more than one subgroup. Source: Substance Abuse and Mental Health Services Administration (11)

b NA indicates not applicable; values are too small to report.
